# 1-(2-Hy­droxy-2-phenyl­eth­yl)-3-(4-meth­oxy­phen­yl)urea

**DOI:** 10.1107/S1600536811036464

**Published:** 2011-09-14

**Authors:** Hyeong Choi, Yong Suk Shim, Sung Chun Lee, Sung Kwon Kang, Chang Keun Sung

**Affiliations:** a Department of Chemistry, Chungnam National University, Daejeon 305-764, Republic of Korea; b Department of Food Science and Technology, Chungnam National University, Daejeon 305-764, Republic of Korea

## Abstract

In the title compound, C_16_H_18_N_2_O_3_, the dihedral angle between the 4-meth­oxy­phenyl ring and the urea group is 35.6 (2) °. The H atoms of the urea NH groups are positioned *syn* to each other. In the crystal, inter­molecular N—H⋯O and O—H⋯O hydrogen bonds link the mol­ecules into a two-dimensional array in the *ac* plane; the carbonyl-O atom is trifurcated.

## Related literature

For general background to melanin, see: Prota (1988 ▶). For the development of potent inhibitory agents of tyrosinase, see: Khan *et al.* (2006 ▶); Kojima *et al.* (1995 ▶); Cabanes *et al.* (1994 ▶); Son *et al.* (2000 ▶); Iida *et al.* (1995 ▶).
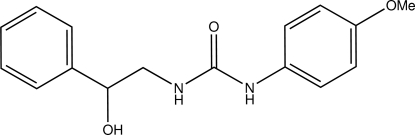


## Experimental

### 

#### Crystal data

C_16_H_18_N_2_O_3_
*M* *_r_* = 286.32
Monoclinic, 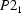

*a* = 6.8120 (6) Å
*b* = 8.7659 (7) Å
*c* = 12.1393 (10) Å
β = 97.009 (3)°
*V* = 719.46 (10) Å^3^
*Z* = 2
Mo *K*α radiation
μ = 0.09 mm^−1^
*T* = 296 K
0.07 × 0.05 × 0.03 mm

#### Data collection

Bruker SMART CCD area-detector diffractometer
5017 measured reflections
1947 independent reflections
1782 reflections with *I* > 2σ(*I*)
*R*_int_ = 0.073

#### Refinement

*R*[*F* ^2^ > 2σ(*F* ^2^)] = 0.060
*wR*(*F* ^2^) = 0.166
*S* = 1.05
1947 reflections
201 parameters
1 restraint
H atoms treated by a mixture of independent and constrained refinement
Δρ_max_ = 0.71 e Å^−3^
Δρ_min_ = −0.26 e Å^−3^

### 

Data collection: *SMART* (Bruker, 2002 ▶); cell refinement: *SAINT* (Bruker, 2002 ▶); data reduction: *SAINT*; program(s) used to solve structure: *SHELXS97* (Sheldrick, 2008 ▶); program(s) used to refine structure: *SHELXL97* (Sheldrick, 2008 ▶); molecular graphics: *ORTEP-3 for Windows* (Farrugia, 1997 ▶); software used to prepare material for publication: *WinGX* publication routines (Farrugia, 1999 ▶).

## Supplementary Material

Crystal structure: contains datablock(s) global, I. DOI: 10.1107/S1600536811036464/tk2785sup1.cif

Structure factors: contains datablock(s) I. DOI: 10.1107/S1600536811036464/tk2785Isup2.hkl

Supplementary material file. DOI: 10.1107/S1600536811036464/tk2785Isup3.cml

Additional supplementary materials:  crystallographic information; 3D view; checkCIF report

## Figures and Tables

**Table 1:** Hydrogen-bond geometry (Å, °)

*D*—H⋯*A*	*D*—H	H⋯*A*	*D*⋯*A*	*D*—H⋯*A*
O8—H8⋯O12^i^	0.86 (7)	2.09 (7)	2.863 (5)	150 (6)
N10—H10⋯O12^ii^	0.81 (6)	2.35 (6)	3.098 (5)	153 (5)
N13—H13⋯O12^ii^	0.79 (5)	2.14 (5)	2.898 (5)	161 (4)

Symmetry codes: (i) 
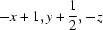
; (ii) 
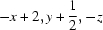
.

**Fig. 1.**
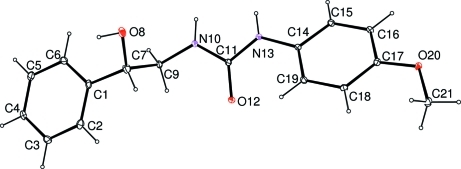
Molecular structure of (I), showing the atom-numbering scheme and 30% probability ellipsoids.

**Fig. 2.**
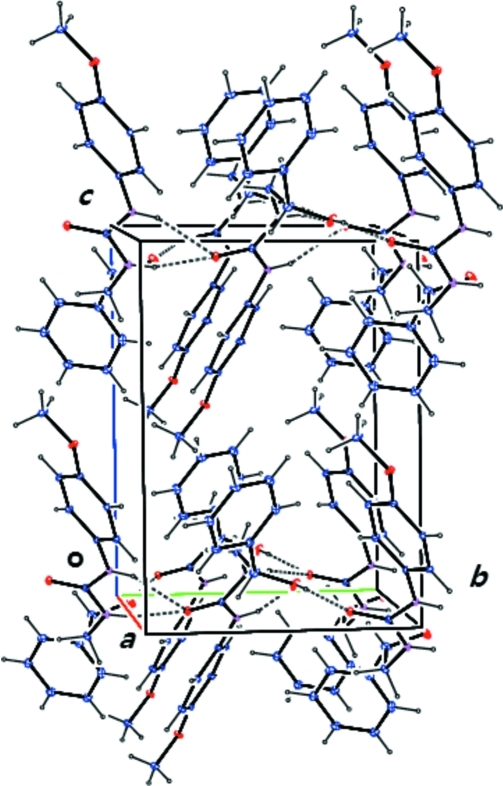
Part of the crystal structure of (I), showing 2-D array of molecules linked by intermolecular N—H···O and O—H···O hydrogen bonds (dashed lines).

## supplementary crystallographic information

### Comment

Melanin is one of the most widely distributed pigments and is found in bacteria, fungi, plants and animals. It is a heterogeneous polyphenol-like biopolymer with a complex structure and colour varying from yellow to black (Prota, 1988). Tyrosinase inhibitors are clinically useful for the treatment of some dermatological disorders associated with melanin hyperpigmentation and are also important in the cosmetic industry for whitening and depigmentation after sunburn (Khan *et al.*, 2006). Numerous potential tyrosinase inhibitors have been discovered from natural and synthetic sources, such as ascorbic acid (Kojima *et al.*, 1995), kojic acid (Cabanes *et al.*, 1994), and tropolone (Son *et al.*, 2000; Iida *et al.*, 1995). But some of their individual activities are either not potent enough to be considered of practical use or not compatible with safety regulations for food and cosmetic additives. In our continuing search for tyrosinase inhibitors, we have synthesized the title compound, (I), from the reaction of 2-amino-1-phenylethanol and 4-methoxyphenyl isocyanate under ambient conditions. Herein, the crystal structure of (I) is described (Fig. 1).

The 4-methoxyphenyl unit is almost planar, with an r.m.s. deviation of 0.031 Å from the least-squares plane defined by the eight constituent atoms. The dihedral angle between the 4-methoxyphenyl ring and the urea plane is 35.6 (2) °. The H atoms of the urea NH groups are positioned *syn* to each other. The presence of intermolecular N—H···O and O—H···O hydrogen bonds link the molecules into a two-dimensional array in the *ac* plane (Fig. 2, Table 1). The urea-O accepts three hydrogen bonds, one from –OH and two from –NH groups.

### Experimental

2-Amino-1-phenylethanol and 4-methoxyphenyl isocyanate were purchased from Sigma Chemical Co. All other chemicals and solvents were of analytical grade and were used without further purification. The title compound (I) was prepared from the reaction of 2-amino-1-phenylethanol (0.3 g, 1.2 mmol) with 4-methoxyphenyl isocyanate (0.39 g, 1.0 mmol) in acetonitrile (6 ml) with stirring. The reaction was completed within 1 h at room temperature. The solvents were removed under reduced pressure, collected and washed with dichloromethane. Removal of the solvent gave a white solid (84%; *M*.pt 468 K). Colourless crystals of (I) were obtained from its ethanolic solution by slow evaporation of the solvent at room temperature.

### Refinement

The NH H atoms were located in a difference Fourier map and refined freely. The OH H atom was located in a difference Fourier map and refined with *U*_iso_(H) = 1.2*U*_eq_(O). The remaining H atoms were positioned geometrically and refined using a riding model with C—H = 0.93–0.97 Å, and with *U*_iso_(H) = 1.2*U*_eq_ (C) for aromatic and methylene, and 1.5*U*_eq_(C) for methyl H atoms. In the absence of significant anomalous scattering effects, 578 Friedel pairs were averaged in the final refinement. The maximum and minimum residual electron density peaks of 0.71 and -0.26 eÅ^-3^, respectively, were located at 0.99 Å and 0.42 Å from the C7 and H7 atoms, respectively.

### Crystal data

**Table:**

C_16_H_18_N_2_O_3_	*F*(000) = 304
*M**_r_* = 286.32	*D*_x_ = 1.322 Mg m^−^^3^
Monoclinic, *P*2_1_	Mo *K*α radiation, λ = 0.71073 Å
Hall symbol: P 2yb	Cell parameters from 4535 reflections
*a* = 6.8120 (6) Å	θ = 2.9–28.1°
*b* = 8.7659 (7) Å	µ = 0.09 mm^−^^1^
*c* = 12.1393 (10) Å	*T* = 296 K
β = 97.009 (3)°	Block, colourless
*V* = 719.46 (10) Å^3^	0.07 × 0.05 × 0.03 mm
*Z* = 2	

### Data collection

**Table:**

Bruker SMART CCD area-detector diffractometer	*R*_int_ = 0.073
graphite	θ_max_ = 25.5°, θ_min_ = 1.7°
φ and ω scans	*h* = −7→8
5017 measured reflections	*k* = −5→10
1947 independent reflections	*l* = −7→14
1782 reflections with *I* > 2σ(*I*)	

### Refinement

**Table:**

Refinement on *F*^2^	Primary atom site location: structure-invariant direct methods
Least-squares matrix: full	Secondary atom site location: difference Fourier map
*R*[*F*^2^ > 2σ(*F*^2^)] = 0.060	Hydrogen site location: inferred from neighbouring sites
*wR*(*F*^2^) = 0.166	H atoms treated by a mixture of independent and constrained refinement
*S* = 1.05	*w* = 1/[σ^2^(*F*_o_^2^) + (0.0819*P*)^2^ + 0.6912*P*] where *P* = (*F*_o_^2^ + 2*F*_c_^2^)/3
1947 reflections	(Δ/σ)_max_ < 0.001
201 parameters	Δρ_max_ = 0.71 e Å^−^^3^
1 restraint	Δρ_min_ = −0.26 e Å^−^^3^

### Special details

**Table:**

Geometry. All s.u.'s (except the s.u. in the dihedral angle between two l.s. planes) are estimated using the full covariance matrix. The cell s.u.'s are taken into account individually in the estimation of s.u.'s in distances, angles and torsion angles; correlations between s.u.'s in cell parameters are only used when they are defined by crystal symmetry. An approximate (isotropic) treatment of cell s.u.'s is used for estimating s.u.'s involving l.s. planes.
Refinement. Refinement of *F*^2^ against ALL reflections. The weighted *R*-factor *wR* and goodness of fit *S* are based on *F*^2^, conventional *R*-factors *R* are based on *F*, with *F* set to zero for negative *F*^2^. The threshold expression of *F*^2^ > 2σ(*F*^2^) is used only for calculating *R*-factors(gt) *etc*. and is not relevant to the choice of reflections for refinement. *R*-factors based on *F*^2^ are statistically about twice as large as those based on *F*, and *R*- factors based on ALL data will be even larger.

### Fractional atomic coordinates and isotropic or equivalent isotropic displacement parameters (Å^2^)

**Table:**

	*x*	*y*	*z*	*U*_iso_*/*U*_eq_	
C1	0.3090 (6)	0.9178 (6)	−0.2016 (4)	0.0461 (12)	
C2	0.2104 (7)	0.7832 (7)	−0.1959 (4)	0.0533 (14)	
H2	0.2401	0.7201	−0.1345	0.064*	
C3	0.0666 (8)	0.7396 (7)	−0.2805 (4)	0.0542 (13)	
H3	0.0017	0.6469	−0.2758	0.065*	
C4	0.0191 (7)	0.8310 (7)	−0.3707 (4)	0.0505 (13)	
H4	−0.0787	0.8012	−0.4268	0.061*	
C5	0.1151 (7)	0.9660 (7)	−0.3783 (4)	0.0530 (14)	
H5	0.0838	1.0293	−0.4394	0.064*	
C6	0.2616 (7)	1.0081 (7)	−0.2929 (5)	0.0571 (14)	
H6	0.3285	1.0998	−0.2983	0.069*	
C7	0.4712 (7)	0.9574 (7)	−0.1086 (5)	0.0613 (16)	
H7	0.4519	0.8924	−0.0451	0.074*	
O8	0.4713 (6)	1.1060 (5)	−0.0726 (3)	0.0631 (11)	
H8	0.347 (10)	1.118 (8)	−0.071 (5)	0.076*	
C9	0.6694 (6)	0.9180 (6)	−0.1428 (3)	0.0378 (10)	
H9A	0.6796	0.8079	−0.1485	0.045*	
H9B	0.6782	0.9604	−0.2159	0.045*	
N10	0.8347 (5)	0.9736 (5)	−0.0668 (3)	0.0369 (9)	
H10	0.876 (8)	1.060 (7)	−0.072 (4)	0.052 (17)*	
C11	0.9310 (5)	0.8900 (5)	0.0159 (3)	0.0309 (9)	
O12	0.8885 (4)	0.7545 (3)	0.0342 (2)	0.0365 (7)	
N13	1.0818 (5)	0.9640 (5)	0.0765 (3)	0.0345 (8)	
H13	1.108 (7)	1.048 (7)	0.060 (4)	0.036 (14)*	
C14	1.2036 (6)	0.9091 (5)	0.1709 (3)	0.0305 (9)	
C15	1.3978 (6)	0.9599 (5)	0.1905 (3)	0.0351 (10)	
H15	1.4482	1.0223	0.1387	0.042*	
C16	1.5166 (6)	0.9188 (5)	0.2859 (3)	0.0349 (10)	
H16	1.6459	0.9546	0.2983	0.042*	
C17	1.4460 (6)	0.8252 (5)	0.3630 (3)	0.0345 (10)	
C18	1.2540 (6)	0.7706 (6)	0.3442 (3)	0.0383 (10)	
H18	1.2069	0.705	0.3951	0.046*	
C19	1.1310 (6)	0.8140 (5)	0.2487 (3)	0.0369 (10)	
H19	1.001	0.7795	0.237	0.044*	
O20	1.5764 (4)	0.7926 (4)	0.4554 (2)	0.0435 (9)	
C21	1.5099 (8)	0.6908 (7)	0.5350 (4)	0.0520 (13)	
H21A	1.6131	0.6768	0.5954	0.078*	
H21B	1.4764	0.5942	0.5005	0.078*	
H21C	1.3954	0.7331	0.5626	0.078*	

### Atomic displacement parameters (Å^2^)

**Table:**

	*U*^11^	*U*^22^	*U*^33^	*U*^12^	*U*^13^	*U*^23^
C1	0.029 (2)	0.051 (3)	0.057 (3)	0.014 (2)	0.0003 (18)	−0.010 (3)
C2	0.045 (3)	0.067 (4)	0.048 (2)	0.015 (3)	0.010 (2)	0.003 (3)
C3	0.042 (3)	0.054 (3)	0.069 (3)	−0.007 (2)	0.016 (2)	0.004 (3)
C4	0.029 (2)	0.071 (4)	0.049 (2)	−0.004 (2)	−0.0010 (18)	−0.006 (3)
C5	0.041 (3)	0.062 (4)	0.056 (3)	0.013 (3)	0.008 (2)	0.014 (3)
C6	0.035 (3)	0.044 (3)	0.094 (4)	0.000 (2)	0.015 (3)	−0.002 (3)
C7	0.044 (3)	0.070 (4)	0.067 (3)	0.006 (3)	−0.002 (2)	−0.025 (3)
O8	0.0414 (18)	0.065 (3)	0.080 (2)	0.0104 (19)	−0.0049 (17)	−0.033 (2)
C9	0.029 (2)	0.040 (3)	0.042 (2)	0.0025 (19)	−0.0037 (16)	−0.006 (2)
N10	0.0314 (19)	0.034 (2)	0.0427 (19)	−0.0011 (17)	−0.0043 (14)	−0.0009 (18)
C11	0.0236 (18)	0.033 (2)	0.0371 (19)	0.0026 (17)	0.0090 (15)	−0.0025 (19)
O12	0.0340 (15)	0.0310 (18)	0.0438 (15)	−0.0032 (13)	0.0016 (11)	−0.0008 (13)
N13	0.0353 (19)	0.031 (2)	0.0355 (17)	−0.0055 (17)	−0.0031 (14)	0.0057 (17)
C14	0.030 (2)	0.030 (2)	0.0311 (18)	−0.0019 (18)	0.0014 (15)	−0.0015 (18)
C15	0.037 (2)	0.037 (3)	0.0305 (18)	−0.006 (2)	0.0044 (15)	−0.0015 (19)
C16	0.031 (2)	0.039 (3)	0.0342 (19)	−0.0056 (19)	0.0017 (15)	−0.0048 (19)
C17	0.039 (2)	0.035 (2)	0.0292 (18)	0.004 (2)	0.0013 (16)	−0.0045 (19)
C18	0.038 (2)	0.043 (3)	0.0347 (19)	−0.005 (2)	0.0052 (16)	0.007 (2)
C19	0.029 (2)	0.040 (3)	0.041 (2)	−0.0039 (19)	0.0045 (16)	0.000 (2)
O20	0.0412 (17)	0.049 (2)	0.0375 (15)	−0.0036 (15)	−0.0045 (12)	0.0146 (15)
C21	0.049 (3)	0.065 (4)	0.041 (2)	−0.003 (3)	−0.001 (2)	0.021 (2)

### Geometric parameters (Å, °)

**Table:**

C1—C2	1.363 (8)	N10—H10	0.81 (6)
C1—C6	1.369 (7)	C11—O12	1.249 (5)
C1—C7	1.520 (7)	C11—N13	1.354 (5)
C2—C3	1.384 (7)	N13—C14	1.415 (5)
C2—H2	0.93	N13—H13	0.79 (5)
C3—C4	1.364 (8)	C14—C15	1.389 (5)
C3—H3	0.93	C14—C19	1.395 (6)
C4—C5	1.361 (8)	C15—C16	1.377 (6)
C4—H4	0.93	C15—H15	0.93
C5—C6	1.398 (7)	C16—C17	1.375 (6)
C5—H5	0.93	C16—H16	0.93
C6—H6	0.93	C17—O20	1.374 (4)
C7—O8	1.374 (7)	C17—C18	1.385 (6)
C7—C9	1.501 (7)	C18—C19	1.398 (6)
C7—H7	0.98	C18—H18	0.93
O8—H8	0.86 (7)	C19—H19	0.93
C9—N10	1.450 (5)	O20—C21	1.429 (6)
C9—H9A	0.97	C21—H21A	0.96
C9—H9B	0.97	C21—H21B	0.96
N10—C11	1.347 (5)	C21—H21C	0.96
			
C2—C1—C6	118.2 (4)	C9—N10—H10	121 (4)
C2—C1—C7	118.5 (5)	O12—C11—N10	123.2 (4)
C6—C1—C7	123.2 (5)	O12—C11—N13	122.4 (4)
C1—C2—C3	120.7 (5)	N10—C11—N13	114.4 (4)
C1—C2—H2	119.7	C11—N13—C14	127.5 (4)
C3—C2—H2	119.7	C11—N13—H13	119 (3)
C4—C3—C2	120.7 (5)	C14—N13—H13	113 (3)
C4—C3—H3	119.6	C15—C14—C19	119.0 (3)
C2—C3—H3	119.6	C15—C14—N13	118.8 (4)
C5—C4—C3	119.8 (5)	C19—C14—N13	122.0 (3)
C5—C4—H4	120.1	C16—C15—C14	120.7 (4)
C3—C4—H4	120.1	C16—C15—H15	119.7
C4—C5—C6	119.0 (5)	C14—C15—H15	119.7
C4—C5—H5	120.5	C17—C16—C15	120.7 (4)
C6—C5—H5	120.5	C17—C16—H16	119.7
C1—C6—C5	121.6 (5)	C15—C16—H16	119.7
C1—C6—H6	119.2	O20—C17—C16	115.8 (4)
C5—C6—H6	119.2	O20—C17—C18	124.5 (4)
O8—C7—C9	109.9 (5)	C16—C17—C18	119.7 (3)
O8—C7—C1	115.1 (4)	C17—C18—C19	120.1 (4)
C9—C7—C1	109.8 (4)	C17—C18—H18	120
O8—C7—H7	107.2	C19—C18—H18	120
C9—C7—H7	107.2	C14—C19—C18	119.8 (4)
C1—C7—H7	107.2	C14—C19—H19	120.1
C7—O8—H8	100 (5)	C18—C19—H19	120.1
N10—C9—C7	113.6 (4)	C17—O20—C21	117.1 (3)
N10—C9—H9A	108.8	O20—C21—H21A	109.5
C7—C9—H9A	108.8	O20—C21—H21B	109.5
N10—C9—H9B	108.8	H21A—C21—H21B	109.5
C7—C9—H9B	108.8	O20—C21—H21C	109.5
H9A—C9—H9B	107.7	H21A—C21—H21C	109.5
C11—N10—C9	124.1 (4)	H21B—C21—H21C	109.5
C11—N10—H10	115 (4)		

### Hydrogen-bond geometry (Å, °)

**Table:**

*D*—H···*A*	*D*—H	H···*A*	*D*···*A*	*D*—H···*A*
O8—H8···O12^i^	0.86 (7)	2.09 (7)	2.863 (5)	150 (6)
N10—H10···O12^ii^	0.81 (6)	2.35 (6)	3.098 (5)	153 (5)
N13—H13···O12^ii^	0.79 (5)	2.14 (5)	2.898 (5)	161 (4)

Symmetry codes: (i) −*x*+1, *y*+1/2, −*z*; (ii) −*x*+2, *y*+1/2, −*z*.

## References

[bb1] Bruker (2002). *SAINT* and *SMART* Bruker AXS Inc., Madison, Wisconsin, USA.

[bb2] Cabanes, J., Chazarra, S. & Garcia-Carmona, F. (1994). *J. Pharm. Pharmacol.* **46**, 982–985. 10.1111/j.2042-7158.1994.tb03253.x 7714722

[bb3] Farrugia, L. J. (1997). *J. Appl. Cryst.* **30**, 565.

[bb4] Farrugia, L. J. (1999). *J. Appl. Cryst.* **32**, 837–838.

[bb5] Iida, K., Hase, K., Shimomura, K., Sudo, S. & Kadota, S. (1995). *Planta Med.* **61**, 425–428. 10.1055/s-2006-958129 7480203

[bb6] Khan, K. M., Mughal, U. R., Khan, M. T. H., Perveen, S., Ullah, Z. & Choudhary, M. I. (2006). *Bioorg. Med. Chem.* **14**, 344–351. 10.1016/j.bmc.2006.05.014 16750372

[bb7] Kojima, S., Yamaguch, K., Morita, K., Ueno, Y. & Paolo, R. (1995). *Biol. Pharm. Bull.* **18**, 1076–1080. 10.1248/bpb.18.1076 8535399

[bb8] Prota, G. (1988). *Med. Res. Rev.* **8**, 525–556. 10.1002/med.2610080405 3057299

[bb9] Sheldrick, G. M. (2008). *Acta Cryst.* A**64**, 112–122. 10.1107/S0108767307043930 18156677

[bb10] Son, S. M., Moon, K. D. & Lee, C. Y. (2000). *J. Agric. Food Chem.* **48**, 2071–2074. 10.1021/jf991397x 10888500

